# Supplementary material for: Synchronous mid-Holocene marine and terrestrial deglaciation in the Ross Sea, Antarctica
Source: Nat Commun. 2025 Dec 19;16:11291. doi: 10.1038/s41467-025-65494-5 (PMC12722368; doi:10.1038/s41467-025-65494-5)
Supplement: Supplementary file 1 — Supplementary Information [file 41467_2025_65494_MOESM1_ESM.pdf]

Synchronous mid-Holocene marine and terrestrial deglaciation in the Ross Sea, Antarctica

Rebecca L. Parker[1,a], Christina R. Riesselman[1,2], Olivia J. Truax[1,3], Richard S. Jones[4], Jae Il Lee[5], Min Kyung Lee[5], Geraldine Jacobsen[6], Brad E. Rosenheim[7], Cristina Subt[7], Atun Zawadzki[6], Catherine Ginnane[8], Sebastian Naeher[8,9], Gavin Dunbar[10], Robert M. McKay[10], Richard Levy[8], Jocelyn Turnbull[8,11], Kyu-Cheul Yoo[5]

[1]Department of Geology, University of Otago, Dunedin 9054, New Zealand

[2]Department of Marine Science, University of Otago, Dunedin 9054, New Zealand

[3]School of Earth and Environment, University of Canterbury, Christchurch, 8140, New Zealand

[4]Securing Antarctica's Environmental Future, School of Earth, Atmosphere and Environment, Monash University, Victoria, Australia

[5]Korean Polar Research Institute (KOPRI), Incheon 21990, Republic of Korea

[6]Australian Nuclear Science and Technology Organisation (ANSTO), Lucas Heights, NSW, Australia

[7]College of Marine Science, University of South Florida, St. Petersburg, FL, USA

[8]Earth Sciences New Zealand, Lower Hutt, Wellington 5010, New Zealand

[9]Lincoln University, Christchurch, New Zealand

[10]Victoria University, Wellington, New Zealand

[11]CIRES, University of Colorado at Boulder, USA

Present address:

a) Faculty of Environment, Science, and Economy, University of Exeter, Exeter, United Kingdom

Corresponding author email address: r.l.parker@exeter.ac.uk

Supplementary information

The supporting information includes extended downcore data for core sites GC71, 72, 78 and 80 (Figure S1-4), collected on *R/V Araon*, Expedition ANA05B-RS15, 2015, details of each facies at the core sites (Table S1), extended information about lead-210 dating (^{210}Pb) (Supplementary Text 1, Supplementary Figure 5) and organic matter composition of ramped pyrolysis oxidation (RPO) splits (Supplementary Text 2, Supplementary Figure 6 and Supplementary Table 2), extended information about the application of reported reservoir ages in SW Ross Sea region (Supplementary Text 3), recalibrated radiocarbon ages (Supplementary Table 3), modelled ice retreat (Supplementary Figure 7) and relevant glacier surface lowering data (Supplementary Table 4).

Down core data for SW Ross Sea sediment cores

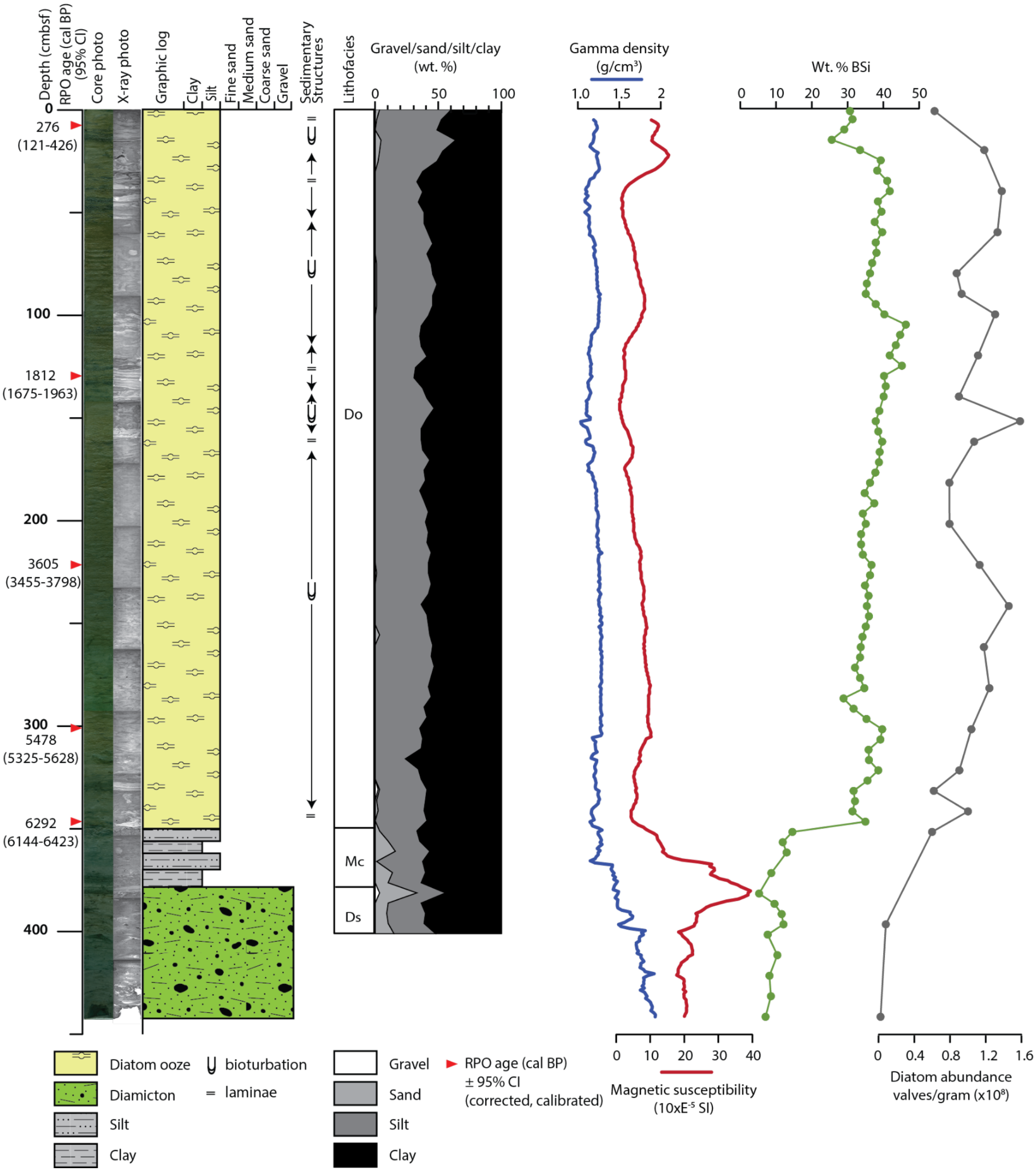

Supplementary Figure 1: Compiled down core data for GC71 (location: 77°5' 10.5288"S, 168°26' 13.3866"E; 967 m depth; 450 cm long), collected from northeast of Ross Island, Ross Sea, Antarctica. Data includes RPO-^{14}C ages, core photo, x-ray image, schematic graphic log, sedimentary structures, facies (Supplementary Table 1), grain size distribution, physical properties of gamma density and magnetic susceptibility, biogenic silica, and absolute diatom abundance. Corrected and calibrated RPO-^{14}C dates of the sediment deposition are denoted by red triangles (Source Data 1&2). Note that dark lines at 25 cm intervals on the x-ray image are edge effect artefacts of individual slab x-rays, which have been stitched together into a continuous image.

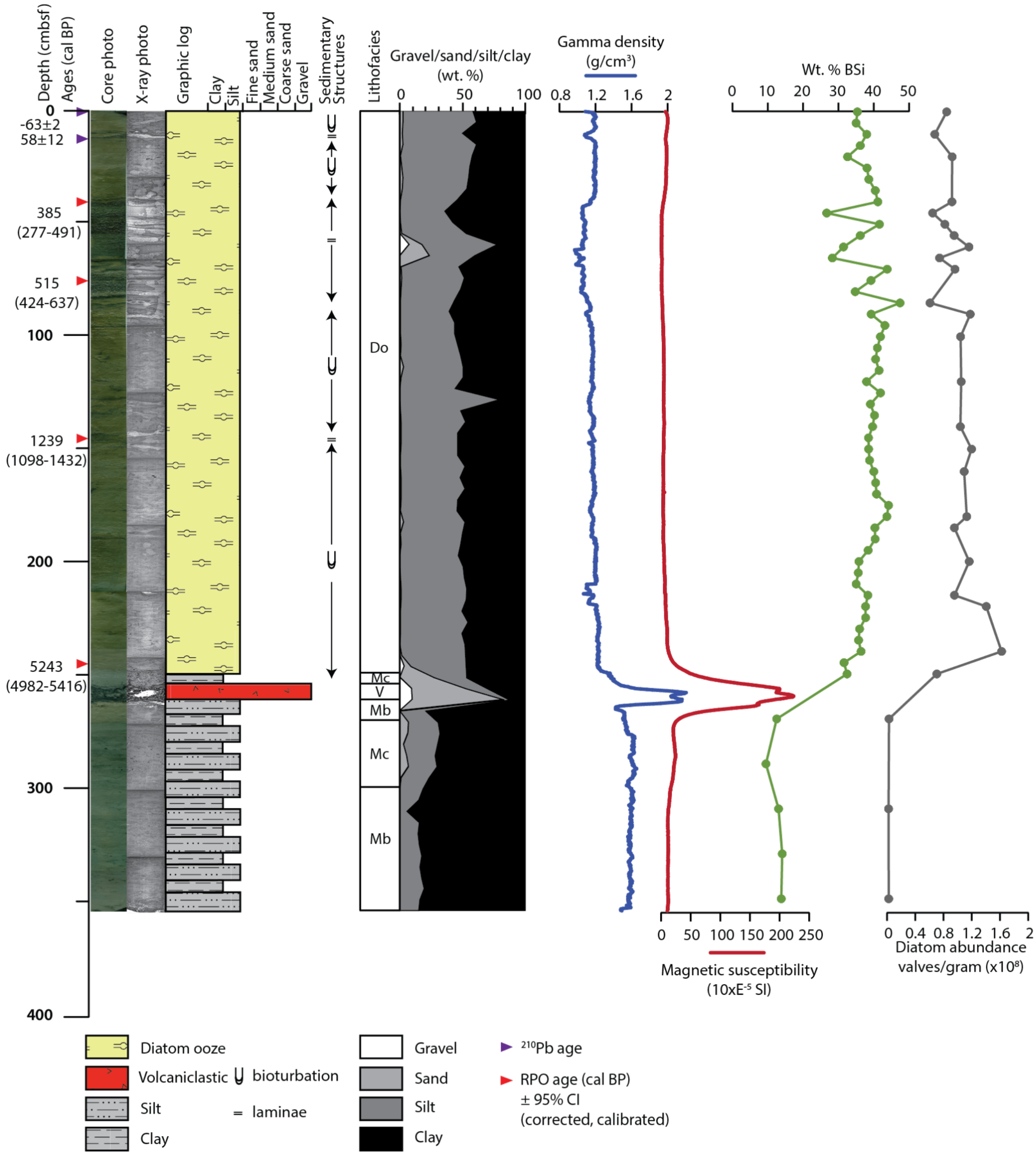

Supplementary Figure 2: Compiled down core data for GC72 (location: 77°14' 43.3314"S, 167°33' 46.944"E; 944 m water depth; 363 cm long), collected from north of Ross Island, Ross Sea, Antarctica. Data includes ^{210}Pb and RPO-^{14}C ages core photo, x-ray image, schematic graphic log, sedimentary structures, facies (Supplementary Table 1), grain size distribution, physical properties of gamma density and magnetic susceptibility, geochemistry of wt. % BSi, and absolute diatom abundance. ^{210}Pb and corrected and calibrated RPO-^{14}C dates of the sediment deposition are denoted by purple and red triangles, respectively (Source Data 1&2). Note that dark lines at 25 cm intervals on the x-ray image are edge effect artefacts of individual slab x-rays, which have been stitched together into a continuous image.

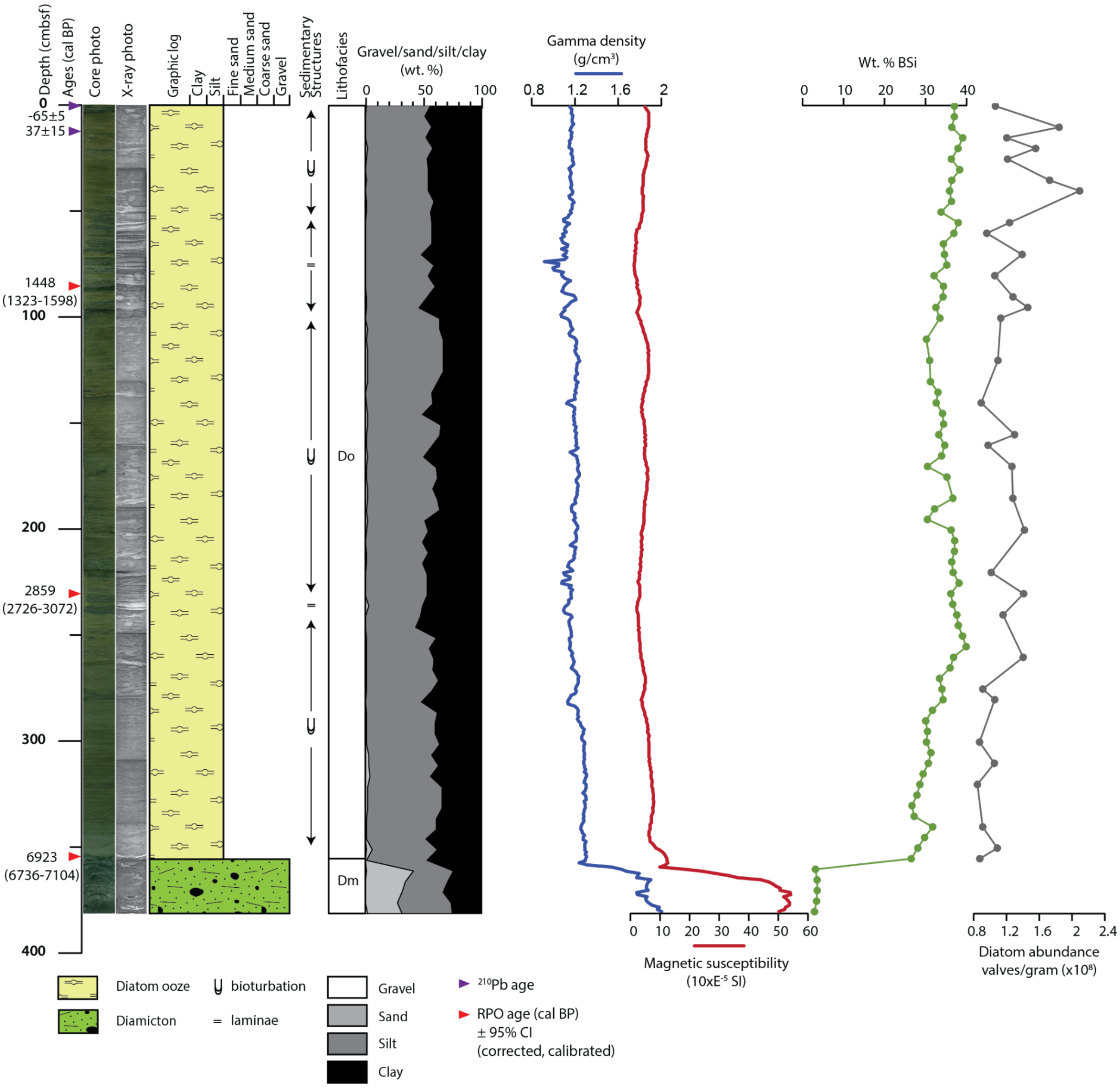

Supplementary Figure 3: Compiled down core data for GC78 (location: 76° 15' 18.9714"S, 163°28' 31.9074"E; 830 m water depth; 393 cm long), collected from offshore of Mawson Glacier, Ross Sea, Antarctica. Data includes ^{210}Pb and RPO-^{14}C ages, core photo, x-ray image, schematic graphic log, sedimentary structures, facies (Supplementary Table 1), grain size distribution, physical properties of gamma density and magnetic susceptibility, geochemistry of wt. % BSi, and absolute diatom abundance. ^{210}Pb and corrected and calibrated RPO-^{14}C dates of sediment deposition are denoted by purple and red triangles, respectively (Source Data 1&2). Note that dark lines at 25 cm intervals on

the x-ray image are edge effect artefacts of individual slab x-rays, which have been stitched together into a continuous image.

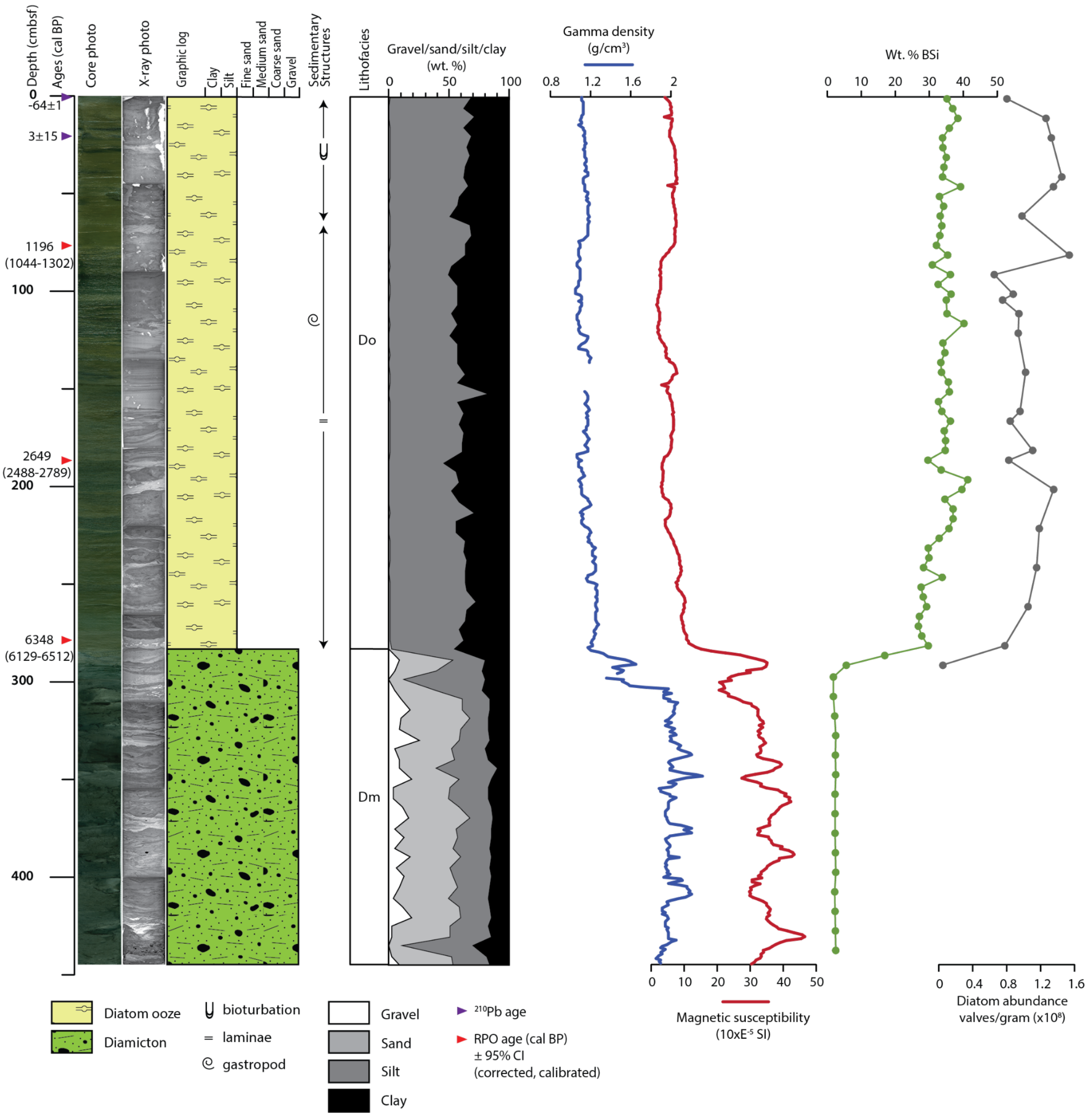

Supplementary Figure 4: Compiled down core data for GC80 (location: 76°48' 12.006"S, 163° 55' 38.8914"E; 732 m water depth; 444 cm long), collected from offshore of McKay Glacier, Ross Sea, Antarctica. Data includes ^{210}Pb and RPO-^{14}C ages, core photo, x-ray image, schematic graphic log, sedimentary structures, facies (Supplementary Table 1), physical properties of gamma density and magnetic susceptibility, geochemistry of wt. % BSi, and absolute diatom abundance. ^{210}Pb and corrected and calibrated RPO ^{14}C dates of sediment deposition are denoted by purple and red triangles, respectively (Source Data 1&2). Note that dark lines at 25 cm intervals on the x-ray image are edge effect artefacts of individual slab x-rays, which have been stitched together into a continuous image.

Facies descriptions

Core descriptions, physical properties, fossil content and x-ray images show a facies succession that is consistent with a glacial retreat model that is well-established for the Ross Sea and shown in Supplementary Table 1 (modified from ref. [1]).

Supplementary Table 1: Facies descriptions and interpretations, modified from ref. [1].

Facies	X-radiograph	Lithology and sedimentary features	Fossil content	Interpretation
**Do**	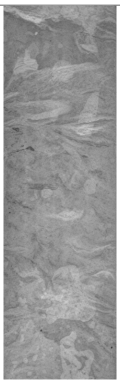 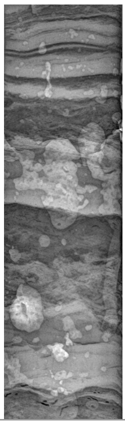	Diatom ooze with bioturbated and laminated intervals. Some clasts present and high wt. % BSi (20-50%). Low magnetic susceptibility (5-20 SI) and gamma density (1-1.3 g/cm^3).	Abundant diatoms (0.4-2 x10^8 valves/gram)	Pelagic sedimentation with ice-rafted debris. General lack of terrigenous material. The presence of clasts suggests open water with ice rafting, which must have existed at numerous times through the Holocene. Bioturbated intervals indicate significant infaunal colonisation, while laminated intervals suggest periods of relatively high productivity (sedimentation rates). An open marine interpretation for this facies is consistent with interpretations reported for similar sediments sampled across the Ross Sea and on other Antarctic continental shelves (e.g. [1,2]).
**Mc**	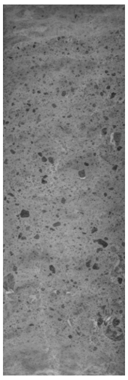	Bioturbated silty clay, grey to green in colour. Clasts present and maybe clay pellets in intervals. Moderate wt. % BSi (5-15%). Varying magnetic susceptibility (5-50 SI) and gamma density (1-1.5 g/ cm^3).	Moderate abundance of diatoms (0.2-1.2 x10^8 valves/gram)	Hemipelagic suspension settling. Bioturbation is likely the result of decreased sediment supply as the grounding line migrated away from the core site, allowing the establishment of a benthos community. This mud unit indicates an ice shelf setting that is located close to basal debris deposition e.g. near the grounding line or iceberg calving area.

**Mb**	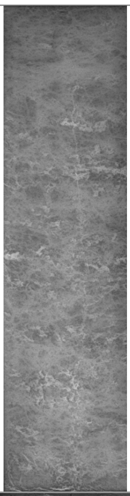	Bioturbated silty clay, grey to green in colour. Structureless with no clasts. Moderate wt. % BSi (10-15%). Moderate magnetic susceptibility (~10 SI) and gamma density (~1.6 g/ cm^3).	Low/moderate abundance of diatoms (0-0.8 x10^8 valves/gram)	Hemipelagic suspension settling. Bioturbation is likely the result of decreased sediment supply as the grounding line migrated away from the core site, allowing the establishment of a benthos community. This mud unit indicates an ice shelf setting that is located away from any basal debris, as there is no coarse ice-rafted debris present.
**Ds**	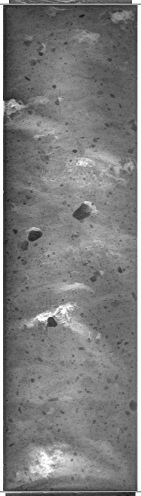	Muddy diamicton. Matrix supported with randomly orientated clasts (>2 mm) and moderate wt. % BSi (<5-15%). Less compacted than Facies Dm. Varying magnetic susceptibility (20-30 SI) and gamma density (1.4-2 g/cm^3).	Trace fragmented diatoms that are reworked (0-0.1 x10^8 valves/gram)	Glaciomarine sediment deposited proximal to the grounding line. The lower sand content than facies Dm suggests some water sorting during deposition. The higher wt. % BSi and less compaction than facies Dm suggests increasing marine influence.
**Dm**	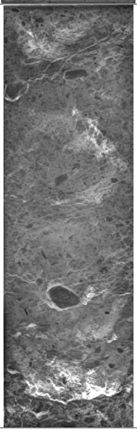	Sandy silt diamicton. Matrix supported with abundant randomly orientated clasts (>2 mm) and low wt. % BSi (<5%). High magnetic susceptibility (~50 SI) and gamma density (1.6-1.8 g/cm^3).	Trace fragmented diatoms that are reworked	Subglacial transport and deposition in a subglacial or grounding zone proximal setting.

Age model

The age models for cores GC71, GC72, GC78, and GC80 are based on accelerator mass spectrometry (AMS) ^{14}C dates and core top ^{210}Pb dates (Source Data 1&2).

Supplementary Text 1: ^{210}Pb dating

Surface sediments represent modern accumulation but are sometimes older due to seafloor erosion or non-deposition. In addition, coring disturbance or over-penetration can remove modern sediments. The ^{210}Pb dating method is used to determine whether core top sediments from the SW Ross Sea record modern (0 years) sedimentation. The downcore ^{210}Pb data is shown in Supplementary Fig. 5 and Source Data 1. Supported ^{210}Pb activity remains relatively constant (~60 Bq/kg) with depth across all cores, while unsupported ^{210}Pb exhibits an exponential decrease with depth, suggesting uniform sedimentation rates (Supplementary Fig. 5). Constant flux constant sedimentation (CFCS) model ages show modern core top ages are obtained for GC72, GC78 and GC80. Calendar age profiles for GC72 and 80 show linear trends, indicating uniform sedimentation rates. GC78 exhibits similar ages in the top 7 cm of sediment, indicating bioturbation. Core GC71 exhibits elevated unsupported ^{210}Pb concentrations (~100 Bq/kg) but lacks the distinct surface peak and exponential decay typically required for reliable ^{210}Pb-based age modelling. While these activity levels are above regional background and suggest the presence of relatively recent sediment, the absence of a clear decay profile prevents us from quantifying accumulation rates or confidently assigning a surface age.

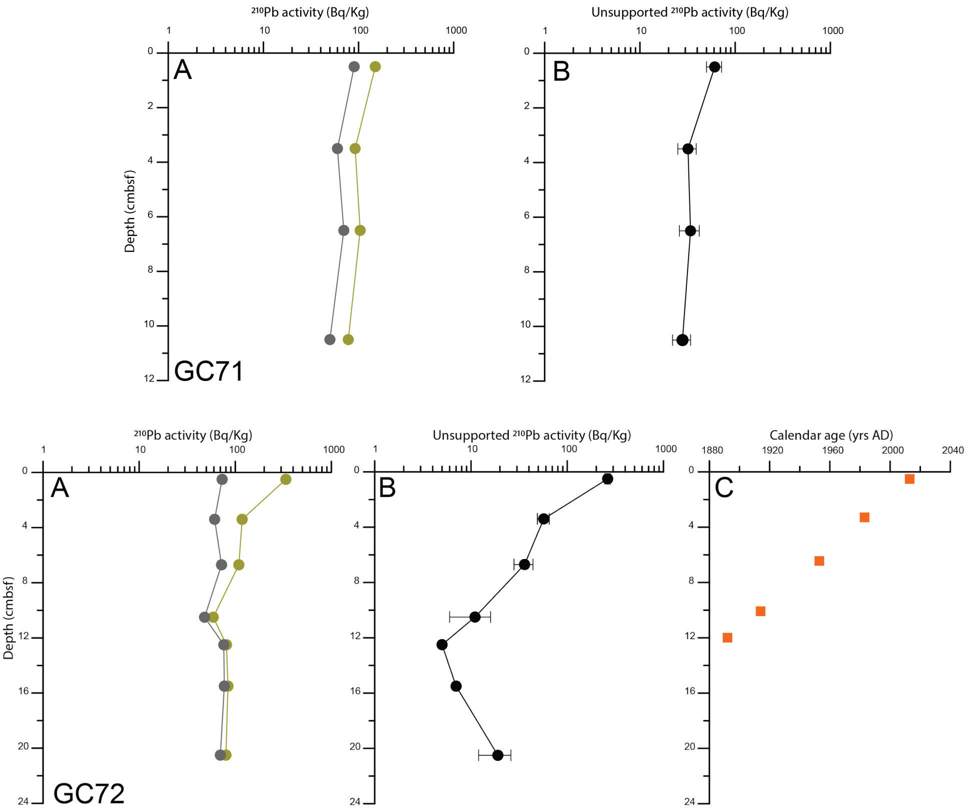

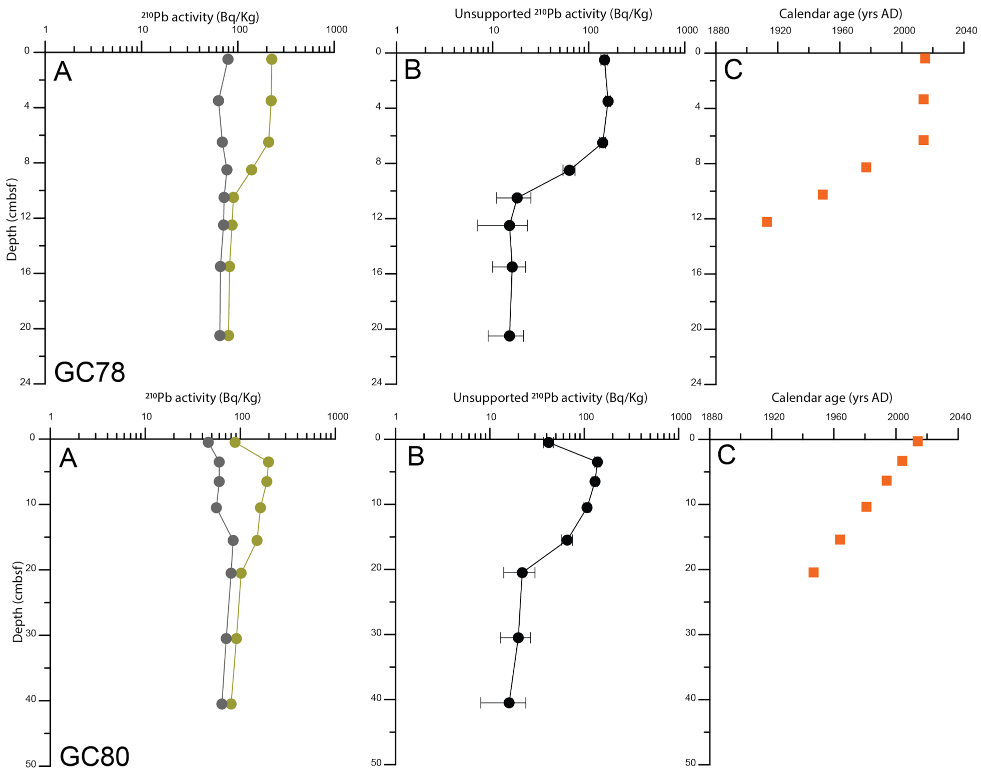

Supplementary Figure 5: GC71, 72, 78 and 80 down core ^{210}Pb dating profiles, where A) Total (green) and supported (grey) ^{210}Pb activity (note the logarithmic scale), B) unsupported ^{210}Pb activity and C) calendar age (yrs AD). Note: GC71 is not suitable for ^{210}Pb age modelling due to the relatively low and lack of decreasing unsupported ^{210}Pb activity profile.

Supplementary Text 2: Organic matter composition of RPO splits revealed by Pyrolysis Gas Chromatography Gas Spectrometry (Py-GC-MS) analysis

RPO-AMS dates are expected to increase in age with increasing pyrolysis temperature[3], because labile, young, syn-depositional sedimentary marine organic matter is predominantly released at lower temperatures. In contrast, more recalcitrant, commonly transported, older, macromolecular organic matter (e.g., kerogen) tends to be evolved at higher temperatures[4]. However, RPO radiocarbon dates from cores GC71, GC72, GC78, and GC80 are often younger in age for the second collected temperature fraction (split 2) than the first fraction (split 1) and exhibit age differences between 16 and 190 ^{14}C yrs BP (Source Data 2). This phenomenon is also observed elsewhere in the Ross Sea[5] and other Antarctic settings[4]. Comparison of RPO and Pyrol data reveals that temperature split 1 can contain volatile, low-molecular weight compounds, which are products of natural degradation of organic matter from a large range of sources and ages[4]. Marine Antarctic sediments are unlikely to contain large terrestrial plant inputs and therefore consist predominantly of either more recent, syn-sedimentary marine organic matter derived from marine phytoplankton or older, transported material derived from marine, terrestrial, or petrogenic sources.

Py-GC-MS results from cores GC71, GC72, and GC80 are interpreted using the literature outlined in Supplementary Table 2. Organic compounds associated with marine primary production (pyrroles, furans, phenols, low molecular weight (especially C_{15}-C_{19} *n*-alkanes)

are more abundant in samples from GC72 and GC80 than core GC71. Pyrroles, compounds derived from pigments in algae, primarily chlorophylls[6,7], are particularly diagnostic of syn-depositional sedimentary marine organic matter and abundant in GC72 and GC80. Aromatic compounds, (e.g. toluene, alkylbenzenes) and sulphur compounds (e.g. thiophenes), may also be derived from marine carbon sources; however, aromatics may also form through degradation of pre-aged, transported organic matter due to their high preservation potential, or are formed by secondary reactions and kerogen cracking during pyrolysis, particularly at higher temperatures[4,8]. Polycyclic aromatic hydrocarbons (PAHs), derived from incomplete combustion of carbon-containing material during pyrolysis, maturation of organic matter, or diagenetic degradation of biogenic precursors[9], are a reliable indicator of refractory carbon sources.

Supplementary Table 2: Summary of compound classes and their approximate interpretation of their sources (e.g., ref. [4] and references therein).

Compound group	Interpretation for an Antarctic marine environment	Dominant origin
Pyrroles	Pigments of primary producers, primarily chlorophylls (e.g., refs. [6,7]).	Autochthonous
Furans	Sugars/polysaccharides from marine phytoplankton (e.g. [10,11] and references therein)	Autochthonous
Phenols	Polysaccharides from marine phytoplankton[12].	Autochthonous
n-Alkanes (and alkenes)	Marine algae and bacteria, particularly in low temperature splits[10]. Also produced by decomposition of high-molecular organic matter and by secondary reactions during pyrolysis[13].	Autochthonous
Toluene and other low molecular weight aromatics	Stable pyrolysis products from a variety of organic matter sources and secondary reactions during pyrolysis[10].	Autochthonous or refractory sources
Thiophenes	Indicators of sulfur in an anoxic water column and sediment, also can be formed during diagenesis and kerogen cracking during pyrolysis[8,14].	Autochthonous or refractory sources
Alkylbenzenes	Decomposition of complex aromatic compounds and secondary reactions during pyrolysis[8].	Autochthonous or refractory sources
Polycyclic aromatic hydrocarbons (PAHs)	Secondary reactions during pyrolysis, maturation of organic matter, or diagenetic degradation of biogenic precursors[9].	Refractory sources

Organic compounds associated with marine primary production are absent in the first temperature fraction of GC71. Instead, split 1 is typically dominated by low-molecular weight alkylbenzenes and toluene, which are ubiquitous, and therefore cannot be used to clearly distinguish autochthonous and allochthonous sources[4,10] (Supplementary Fig. 6). Low-molecular weight compounds as those enriched in the first, low temperature split can form from the degradation of macromolecular, refractory carbon, resulting in higher ages of the first split. Highest abundances of marine-associated compounds such as pyrroles, furans,

phenols, and low-molecular weight *n*-alkanes in the second temperature fraction in GC71 indicates that split 2 most likely contains the highest proportion of autochthonous carbon.

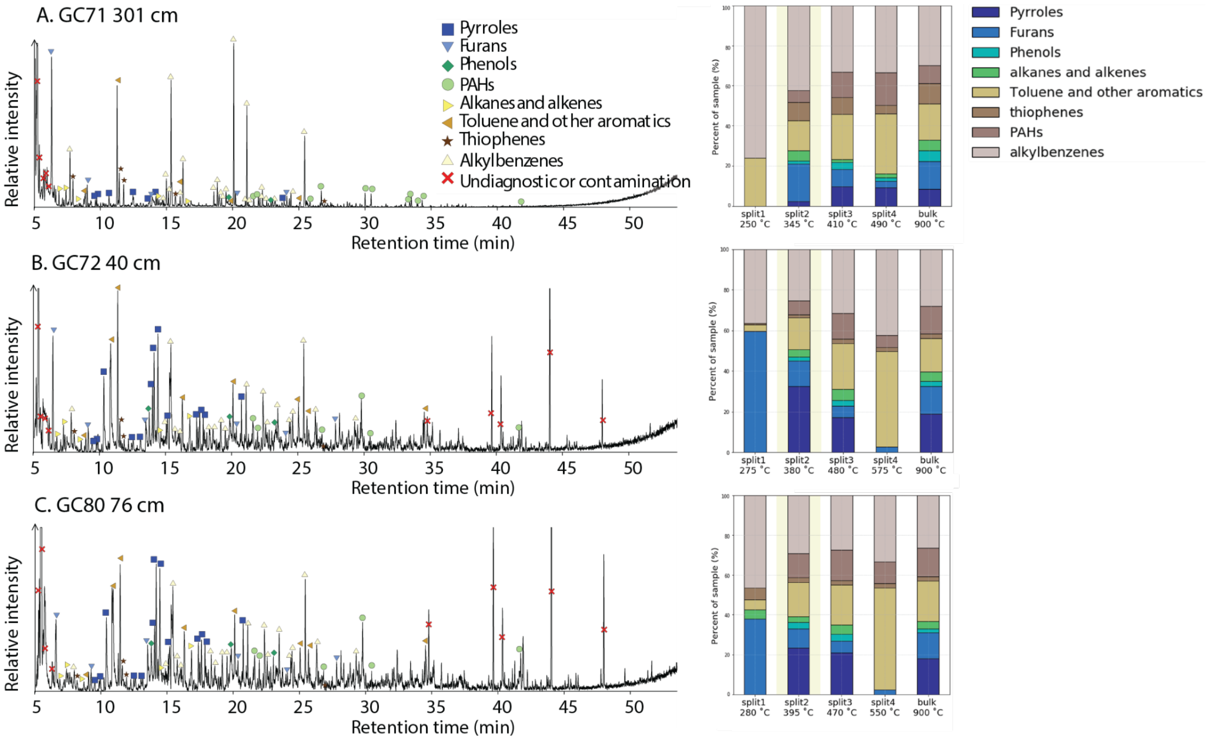

Supplementary Figure 6: Comparison of Py-GC-MS chromatograms from the second temperature splits of A) GC71 301 cm depth, B) GC72 40cm depth, and C) GC80 76 cm depth, alongside bar diagrams summarizing the Py-GC-MS data for all splits of each sample. Symbols highlight peaks attributed to diagnostic compound classes while bar diagrams show the relative abundance of diagnostic compound classes (see Supplementary Table 2). Each bar corresponds to the temperature range for each split, with the minimum temperature indicated.

The proportion of marine-associated organic compounds decreases with increasing maximum temperature in the third and fourth temperature fractions in core GC71 (Supplementary Fig. 6). However, there is a higher proportion of pyrroles, furans, phenols, *n*-alkanes (and alkenes) in split 4 (16% total) in core GC71 compared to samples from cores GC72 and GC80. This is likely a result of the lower maximum temperatures in each fraction in core GC71 compared to the other samples (Supplementary Fig. 6); the temperature range for split 4 for GC71 has substantial overlap with the split 3 temperature range for the other cores. The lower maximum temperature for core GC71 is also relevant to the interpretation of split 1; non-diagnostic, low molecular weight compounds volatilized at a lower temperature (250°C) than all autochthonous marine-associated carbon compounds in core GC71 (Supplementary Fig. 6).

The composition of the first temperature fraction in cores GC72 and GC80 differ from GC71 (Supplementary Fig. 6). Furans, compounds associated with the breakdown of sugars and polysaccharides from phytoplankton, dominate the first temperature fraction in GC72 (60%). Furans (38%) and a smaller proportion of *n*-alkanes and alkenes (5%), which are derived from marine algae, also make up a significant component of the first temperature

fraction in GC80. Although the relative contribution of marine-associated compounds in the first splits is high in GC72 and GC80, both are small (0.07 mg and 0.09 mg, respectively) and the absolute amount of OC obtained is low compared to split 2. Split 2 also contains a more diverse array of marine-associated compounds including phenols, derived from carbohydrates, which is mainly associated with the decay of sugars from phytoplankton, and pyrroles, which are derivatives of chlorophylls and other pigments[6,7,13]. The proportion of marine-associated compounds to non-diagnostic and petrogenic compounds decreases slightly in split 3 and marine compounds of syn-depositional sedimentary material are largely absent in split 4.

Results from Py-GC-MS provide useful context to interpret the discrepancies between split 1 and 2 ages at other depths in the cores (Supplementary Table 2; Source Data 1&2), despite minor differences in RPO methodology at Rafter Radiocarbon Lab (RRL) and the University of South Florida (USF). As discussed above, the composition of RPO temperature fractions, and by extension ^{14}C dates, are sensitive to the collected temperature range of each split. The maximum temperature of the first split for samples analysed at RRL (250-300°C˚, mean 275°C) is lower than the maximum temperature of split 1 for samples analysed at USF (284-345°C, mean 306°C) (Supplementary Fig. 6). Split 2 is also isolated over a narrower temperature range at USF (mean maximum temperature of 358°C) than at RRL (mean maximum temperature of 374°C). These differences arise because the USF laboratory selects cutoff points for each split based on both observations of CO_2 evolution and the mass of each sample to ensure a dateable quantity of material; at RRL the cutoff points are selected based on CO_2 evolution alone, which yields a lower mass of material in the first split and a larger mass for the second split (Supplementary Fig. 6). The first split from the same depths analysed at RRL isolates the lowest temperature fraction in split 1; the second split incorporates organic compounds volatilized across a range of temperatures characteristic of splits 1,2, and 3 isolated at USF. A higher maximum temperature is unlikely to substantially bias the ages of split 2 temperature fractions obtained at RRL because similar compositions between split 2 and split 3 indicate that they contain similar relative proportions of marine-derived carbon.

The low abundance of organic matter in split 1 from GC71 and more diverse array of organic compounds in split 2 from GC72 and GC80 provides convincing evidence that the first RPO temperature fraction from Ross Sea Holocene marine sediment cores can incorporate low-molecular degradation products that make up most lower temperature splits (Supplementary Fig. 6). Based on results from Py-GC-MS, we selected split 2 as reflecting the most accurate maximum age constraint for cores GC71, GC72, GC78, and GC80.

Supplementary Text 3: Reservoir correction and age model development

We developed age-depth models for all sediment cores using a combination of ^{210}Pb and ramped pyrolysis oxidation (RPO) radiocarbon dates, calibrated with the Marine20 calibration curve. In addition to applying the global mean marine reservoir correction inherent to Marine20[15], an additional regional offset (ΔR) is required to account for local oceanographic influences.

In the modern open ocean SW Ross Sea, paired U/Th and ^{14}C dates from exhumed Antarctic corals indicate a ΔR value of 610 ± 120 ^{14}C years[16] (recalculated from [17] for compatibility with Marine20). Similarly, conventional ^{14}C dating of known-age marine specimens and paired ^{14}C -U/Th ages of coral samples from the Ross Sea yields a ΔR value of 609 ± 137 ^{14}C years[18]. Evidence from the coral indicates that the reservoir has been constant during our period of interest, the last 6,000 years[17].

We apply a localized correction to cores GC71 and GC72 to reconcile discrepancies between radiocarbon and ^{210}Pb chronologies. When calibrated using the regional Ross Sea ΔR (609 ± 137 ^{14}C years), the uppermost ^{14}C dates from these cores are significantly older—by 700 and 950 years, respectively—than the ^{210}Pb-based ages, which otherwise indicate modern or near-modern core tops. This, in conjunction with Py-GC-MS results, indicates that RPO did not fully eliminate detrital carbon contamination in these cores. To account for contamination by detrital carbon, we estimated a local contamination offset (LCO) for GC71 and GC72. We reverse-calibrated a paired pre-bomb ^{210}Pb and ^{14}C date from GC72 at 10 cm depth, which yielded a local ΔR of 1480 ± 120 ^{14}C years[19]. Subtracting the regional ΔR (609 ± 137) results in an additional offset of 870 years ± 120 ^{14}C years, which we attribute to some combination of detrital carbon and/or localized variation in ΔR. We incorporated this LCO into the age-depth models for GC71 and GC72 (cf. [20–22]), because no paired pre-bomb ^{210}Pb and ^{14}C date is available for GC71. Although we cannot confidently confirm the presence of a modern core top in GC71 (see ^{210}Pb data), our age model, which includes both the regional ΔR and the LCO, suggests that the core top is statistically indistinguishable from modern (172 cal yr BP, -51 to 361 years, 95% CI).

We calibrated the age models for cores GC78 and 80 using the ΔR value of 609 ± 137 ^{14}C years without an additional LCO. The calibrated radiocarbon ages from the uppermost radiocarbon dates are ~200-300 years older than the corresponding ^{210}Pb dates, suggesting minor detrital carbon contamination. However, because paired ^{14}C and ^{210}Pb dates are post-bomb, the ^{14}C dates may reflect competing influences from older detrital carbon and younger bomb carbon and cannot be reverse-calibrated to Marine20 to reliably diagnose an offset caused solely by detrital carbon and local oceanographic factors[19]. To generate age-depth models for the cores, we excluded the uppermost ^{14}C dates and rely instead on the more precise ^{210}Pb chronology for the surface interval. All other downcore RPO ^{14}C dates are calibrated using the ΔR value of 609 ± 137 ^{14}C years without an additional LCO.

Our age models for cores GC71, 72, 78 and 80 reflect the best available methods for dating Antarctic sediments that lack carbonate. However, they also demonstrate that even RPO-derived ^{14}C dates can be susceptible to detrital carbon contamination. While the use of a constant ΔR value and LCO currently offers the most robust approach to generating chronologies for Holocene Ross Sea sedimentary records, we acknowledge this as an inherent caveat in our analysis.

Supplementary Table 3: Reported and (re)calibrated ^{14}C dates from previous studies that are mentioned in text. Recalibrated ages using Ross Sea-wide ΔR value compiled by [18].

Reference	Sample depth (cm)	Laboratory code	Dated material	Interpretation	Conventional ^{14}C age (yrs BP) ± error	ΔR (yrs) ± error	Calibrated age, yrs (mean)	Age range, yrs (95% CI)
Coulman High, CH-2[1]	50-54	NZA50804	benthic foram	Grounding line retreat	8892 ± 155	609 ± 137	8647	8155-9199
Scott Coast[23]	NA	NA	Modelled beach uplift	Modelled grounding line retreat	7750 ± 150	609 ± 137	7438	6999-7856
Terra Nova Bay[24]	Adelie Cove	TO-5571(4)	Penguin guano		8490 ± 90	609 ± 137	8169	7801-8541
DF80-57[25]	43	AA11876	Reworked shell	Grounding line retreat	7830 ± 60	609 ± 137	7516	7211-7837
Mercer Glacier, Siple Coast[26]		NA	Linear modelling of RPO spectra	Grounding line retreat		1101 ±120*	6300	6200-6400
Bindschadler Ice Stream, Siple Coast[27]	NA	NA	Modelling of Fm values derived from AIO bulk sediment ^{14}C from ref. [28]	Modelled grounding line retreat		NA	1700	1100-4500
Willans Subglacial Lake, Siple Coast[29]	NA	NA	Comparison of measured sediment core porewater ionic concentrations to modelled values using ionic diffusion model.	Modelled grounding line retreat		NA	4300	2400-5800
NE of Ross Island (this study)	GC71	Age-depth model	RPO- bulk sediment	Transition silty clay to diatom ooze – Grounding line retreat	Derived from age-depth model	609 ± 137	7343	7181-7958
N of Ross Island (this study)	GC72	Age-depth model	RPO- bulk sediment	Transition silty clay to diatom ooze – Grounding line retreat	Derived from age-depth model	609 ± 137	6345	6116-7144
Offshore Mawson Glacier (this study)	GC78 356-357	OZU586	RPO- bulk sediment	Transition diamict to diatom ooze – Grounding line retreat	7250 ± 140	609 ± 137	6923	6736-7104

Offshore Mackay Glacier (this study)	GC80	Age-depth model	RPO- bulk sediment	Transition diamict to diatom ooze – Grounding line retreat	Derived from age-depth model	609 ± 137	6467	6276-7279

Calibration of all marine ^{14}C dates generated in BChron package (version 4.7.6[30]) in Rstudio[31] (version 4.3.3). Radiocarbon results were calibrated using the Marine20 calibration curve[15] with the Ross Sea average ΔR of 609 ± 137 ^{14}C years[18]. *Reservoir age derived from measured living amphipods at the grounding zone of the Whillans Ice Stream in 2015[28].

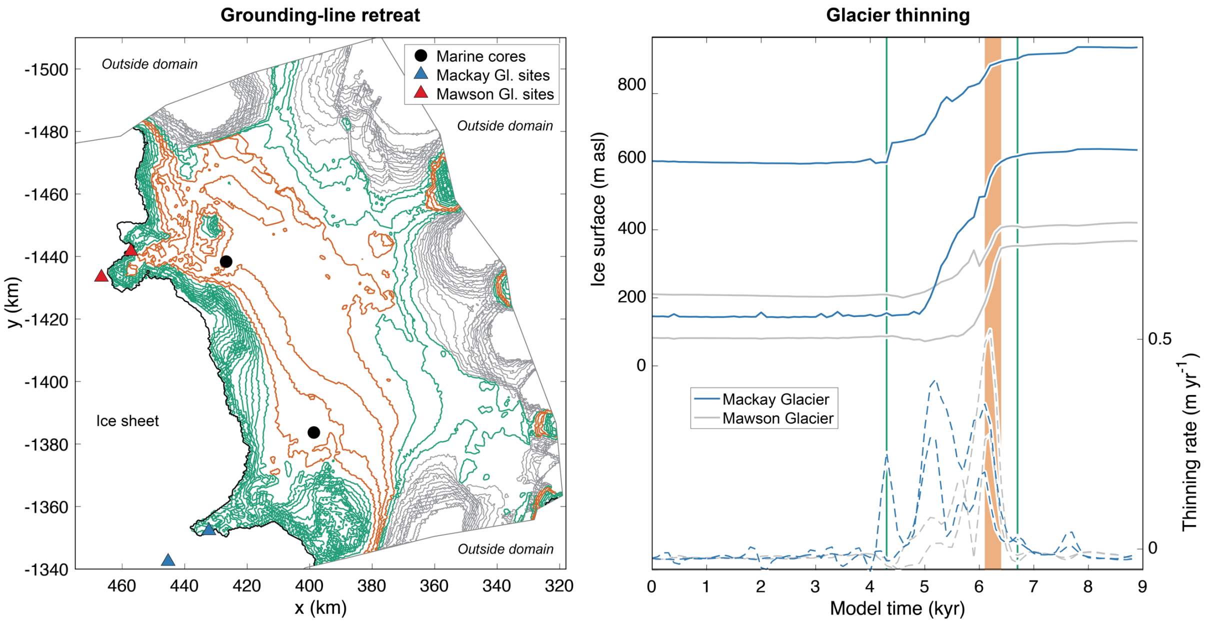

Supplementary Figure 7: Modelled retreat in the SW Ross Sea during the Holocene. The results of an example simulation from an experiment that replicates rapid ice surface lowering recorded at Mackay and Mawson glaciers[32]. The left panel shows the pattern of grounding line retreat in 100-year intervals from the seamounts to the present-day glacier termini (right to left), indicating initially slow retreat which then accelerates into deeper water. The right panel shows the corresponding ice surface lowering at the glacier sites that record rapid thinning (solid lines) and their differentials (dashed lines). The vertical green lines delimit the start and end of modelled rapid thinning, with the grounding lines during this period coloured accordingly in the left panel. The period that the grounding line crossed the cores sites are shown as orange lines (left panel) and an orange bar (right panel). This modelling indicates that retreat at the core sites should capture the middle of the accelerated retreat in the SW Ross Sea and that retreat at these sites was simultaneous with rapid thinning ~40-60 km upstream. In this example, retreat would have coincided with the early part of accelerated glacier surface lowering, with the peak in thinning occurring concurrently (Mawson) or ~1 ka after (Mackay) retreat recorded in the cores.

Supplementary Table 4: Glacier surface lowering from previous studies mentioned in text and figures. Timings of accelerated thinning correspond to when surface lowering was most rapid at each glacier based on the original study. The range in parentheses is an estimate from ref. [33], where available.

**Reference**	**Latitude**	**Longitude**	**Timing of accelerated thinning (ka)**
David Glacier[34]	75.5S	~162-164 E	~7-6
Mackay Glacier[35]	77 S	~162 E	~7.5-5 (7.1-4.9)
Mawson Glacier[36]	76.5 S	~162 E	~7-6
Southern McMurdo Sound[37]	78-78.5 S	~166 E	10.3-7.4
Reedy Glacier[38]	*85.9 S*	*~132 E*	*9-6 (10.3-1.4) (unclear if rapid)*
Beardmore Glacier[39]	~83.5 S	171-172 E	9.3-8 (12.4-7.2)
Scott Glacier[39]	*~85.5 S*	*154 E*	*9.2-3.4 (11.5-1.7) (sustained gradual thinning after 9.2 ka)*
Tucker Glacier[40]	~72.5 S	169-170 E	~13.6-7.5
Aviation Glacier[40]	~74 S	~165 E	~11-7.5
Darwin-Hatherton Glacier system[41]	*~79.5-80 S*	*155-160 E*	*~9-3 (sustained thinning, not rapid)*

References

1. McKay, R. *et al.* Antarctic marine ice-sheet retreat in the Ross Sea during the early Holocene. *Geology* **44**, 7–10 (2016).
2. Domack, E. W., Jacobson, E. A., Shipp, S. & Anderson, J. B. Late Pleistocene–Holocene retreat of the West Antarctic Ice-Sheet system in the Ross Sea: Part 2—Sedimentologic and stratigraphic signature. *Geological Society of America Bulletin* (1999).
3. Rosenheim, B. E. *et al.* Antarctic sediment chronology by programmed-temperature pyrolysis: Methodology and data treatment. *Geochem Geophys Geosyst* **9**, 2007GC001816 (2008).
4. Ginnane, C. E. *et al.* Advancing Antarctic Sediment Chronology through combined ramped pyrolysis oxidation and pyrolysis-GC-MS. *Radiocarbon* 1–20 (2024) doi:10.1017/RDC.2023.116.
5. Truax, O. J. *et al.* Holocene paleoceanographic variability in Robertson Bay, Ross Sea, Antarctica: A marine record of ocean, ice sheet, and climate connectivity. *Quaternary Science Reviews* **332**, 108635 (2024).
6. Jusélius, J. & Sundholm, D. The aromatic pathways of porphins, chlorins and bacteriochlorins. *Phys. Chem. Chem. Phys.* **2**, 2145–2151 (2000).
7. Naeher, S., Schaeffer, P., Adam, P. & Schubert, C. J. Maleimides in recent sediments – Using chlorophyll degradation products for palaeoenvironmental reconstructions. *Geochimica et Cosmochimica Acta* **119**, 248–263 (2013).
8. Saiz-Jimenez, C. The origin of alkylbenzenes and thiophenes in pyrolysates of geochemical samples. *Organic Geochemistry* **23**, 81–85 (1995).
9. Soclo, H. H., Garrigues, P. & Ewald, M. Origin of Polycyclic Aromatic Hydrocarbons (PAHs) in Coastal Marine Sediments: Case Studies in Cotonou (Benin) and Aquitaine (France) Areas. *Marine Pollution Bulletin* **40**, 387–396 (2000).
10. Fabbri, D., Sangiorgi, F. & Vassura, I. Pyrolysis–GC–MS to trace terrigenous organic matter in marine sediments: a comparison between pyrolytic and lipid markers in the Adriatic Sea. *Analytica Chimica Acta* **530**, 253–261 (2005).
11. Maier, K. L. *et al.* Earthquake-triggered submarine canyon flushing transfers young terrestrial and marine organic carbon into the deep sea. *Earth and Planetary Science Letters* **654**, 119241 (2025).
12. Generalić Mekinić, I. *et al.* Phenolic Content of Brown Algae (Pheophyceae) Species: Extraction, Identification, and Quantification. *Biomolecules* **9**, (2019).
13. Chapter 17. Analytical pyrolysis of microorganisms. in *Techniques and Instrumentation in Analytical Chemistry* (ed. Moldoveanu, S. C.) vol. 20 471–483 (Elsevier, 1998).
14. Shawar, L., Grice, K., Holman, A. I. & Amrani, A. Carbon and sulfur isotopic composition of alkyl- and benzo-thiophenes provides insights into their origins and formation pathways. *Organic Geochemistry* **151**, 104163 (2021).
15. Heaton, T. J. *et al.* Marine20—The Marine Radiocarbon Age Calibration Curve (0–55,000 cal BP). *Radiocarbon* **62**, 779–820 (2020).
16. Hall, B. L. *et al.* Widespread southern elephant seal occupation of the Victoria land coast implies a warmer-than-present Ross Sea in the mid-to-late Holocene. *Quaternary Science Reviews* **303**, 107991 (2023).
17. Hall, B. L., Henderson, G. M., Baroni, C. & Kellogg, T. B. Constant Holocene Southern-Ocean 14C reservoir ages and ice-shelf flow rates. *Earth and Planetary Science Letters* **296**, 115–123 (2010).

18. Gao, Y. *et al.* The occupation history of the longest-dwelling Adélie penguin colony reflects Holocene climatic and environmental changes in the Ross Sea, Antarctica. *Quaternary Science Reviews* **284**, 107494 (2022).
19. Stuiver, M. & Reimer, P. J. CALIB rev. 8. *Radiocarbon* **35**, 215–230 (1992).
20. Mezgec, K. *et al.* Holocene sea ice variability driven by wind and polynya efficiency in the Ross Sea. *Nat Commun* **8**, 1334 (2017).
21. Tesi, T. *et al.* Resolving sea ice dynamics in the north-western Ross Sea during the last 2.6 ka: From seasonal to millennial timescales. *Quaternary Science Reviews* **237**, 106299 (2020).
22. Torricella, F. *et al.* Multi-proxy paleoenvironmental reconstruction of Robertson Bay, East Antarctica, since the last glacial period. *Quaternary Science Reviews* **332**, 108629 (2024).
23. Hall, B. L., Baroni, C. & Denton, G. H. Holocene relative sea-level history of the Southern Victoria Land Coast, Antarctica. *Global and Planetary Change* **42**, 241–263 (2004).
24. Baroni, C. & Hall, B. L. A new Holocene relative sea-level curve for Terra Nova Bay, Victoria Land, Antarctica. *J Quaternary Science* **19**, 377–396 (2004).
25. Licht, K. J., Jennings, A. E., Andrews, J. T. & Williams, K. M. Chronology of late Wisconsin ice retreat from the western Ross Sea, Antarctica. *Geol* **24**, 223 (1996).
26. Venturelli, R. A. *et al.* Constraints on the Timing and Extent of Deglacial Grounding Line Retreat in West Antarctica. *AGU Advances* **4**, e2022AV000846 (2023).
27. Neuhaus, S. U. *et al.* Did Holocene climate changes drive West Antarctic grounding line retreat and readvance? *The Cryosphere* **15**, 4655–4673 (2021).
28. Kingslake, J. *et al.* Extensive retreat and re-advance of the West Antarctic Ice Sheet during the Holocene. *Nature* **558**, 430–434 (2018).
29. Venturelli, R. A. *et al.* Mid-Holocene Grounding Line Retreat and Readvance at Whillans Ice Stream, West Antarctica. *Geophysical Research Letters* **47**, e2020GL088476 (2020).
30. Haslett, J. & Parnell, A. A Simple Monotone Process with Application to Radiocarbon-Dated Depth Chronologies. *Journal of the Royal Statistical Society Series C: Applied Statistics* **57**, 399–418 (2008).
31. R Core Team. A Language and Environment for Statistical Computing. R Foundation for Statistical Computing (2024).
32. Jones, R. S., Gudmundsson, G. H., Mackintosh, A. N., McCormack, F. S. & Whitmore, R. J. Ocean-Driven and Topography-Controlled Nonlinear Glacier Retreat During the Holocene: Southwestern Ross Sea, Antarctica. *Geophysical Research Letters* **48**, e2020GL091454 (2021).
33. Small, D., Bentley, M. J., Jones, R. S., Pittard, M. L. & Whitehouse, P. L. Antarctic ice sheet palaeo-thinning rates from vertical transects of cosmogenic exposure ages. *Quaternary Science Reviews* **206**, 65–80 (2019).
34. Stutz, J. *et al.* Mid-Holocene thinning of David Glacier, Antarctica: chronology and controls. *The Cryosphere* **15**, 5447–5471 (2021).
35. Jones, R. S. *et al.* Rapid Holocene thinning of an East Antarctic outlet glacier driven by marine ice sheet instability. *Nat Commun* **6**, 8910 (2015).
36. Jones, R. S. *et al.* Regional-scale abrupt Mid-Holocene ice sheet thinning in the western Ross Sea, Antarctica. *Geology* **49**, 278–282 (2020).
37. Anderson, J. T. H. *et al.* Reconciling marine and terrestrial evidence for post LGM ice sheet retreat in southern McMurdo Sound, Antarctica. *Quaternary Science Reviews* **157**, 1–13 (2017).

38. Todd, C., Stone, J., Conway, H., Hall, B. & Bromley, G. Late Quaternary evolution of Reedy Glacier, Antarctica. *Quaternary Science Reviews* **29**, 1328–1341 (2010).
39. Spector, P. *et al.* Rapid early-Holocene deglaciation in the Ross Sea, Antarctica. *Geophysical Research Letters* **44**, 7817–7825 (2017).
40. Goehring, B. M., Balco, G., Todd, C., Moening-Swanson, I. & Nichols, K. Late-glacial grounding line retreat in the northern Ross Sea, Antarctica. *Geology* **47**, 291–294 (2019).
41. Hillebrand, T. R. *et al.* Holocene thinning of Darwin and Hatherton glaciers, Antarctica, and implications for grounding-line retreat in the Ross Sea. *The Cryosphere* **15**, 3329–3354 (2021).
